# Shape descriptors of the “never resting” microglia in three different acute brain injury models in mice

**DOI:** 10.1186/s40635-015-0039-0

**Published:** 2015-02-24

**Authors:** Elisa R Zanier, Stefano Fumagalli, Carlo Perego, Francesca Pischiutta, Maria-Grazia De Simoni

**Affiliations:** IRCCS – Istituto di Ricerche Farmacologiche Mario Negri, Department of Neuroscience, Via La Masa 19, 20156 Milan, Italy; Department of Anesthesiology and Intensive Care, Neuro ICU, Fondazione IRCCS Cà Granda-Ospedale Maggiore Policlinico, Via F Sforza 35, 20122 Milan, Italy

**Keywords:** Traumatic brain injury, Stroke, Brain ischemia, Microglia, Shape descriptors

## Abstract

**Background:**

The study of microglia and macrophage (M/M) morphology represents a key tool to understand the functional activation state and the pattern of distribution of these cells in acute brain injury. The identification of reliable quantitative morphological parameters is urgently needed to understand these cell roles in brain injury and to explore strategies aimed at therapeutically manipulating the inflammatory response.

**Methods:**

We used three different clinically relevant murine models of focal injury, namely, controlled cortical impact brain injury (traumatic brain injury (TBI)) and transient and permanent occlusion of middle cerebral artery (tMCAo and pMCAo, respectively). Twenty-four hours after injury, M/M cells were labeled by CD11b, and ×40 photomicrographs were acquired by unbiased sampling of the lesion core using a motorized stage microscope. Images were processed with Fiji software to obtain shape descriptors.

**Results:**

We validated several parameters, including area, perimeter, Feret’s diameter (caliper), circularity, aspect ratio, and solidity, providing quantitative information on M/M morphology over wide tissue portions. We showed that the shape descriptors that best represent M/M ramification/elongation are area and perimeter, while circularity and solidity provide information on the ameboid shape. We also provide evidence of the involvement of different populations in local inflammatory events, with macrophages replacing microglia into the lesion core when reperfusion does not occur. Analysis of CD45^high^+ cell morphology, whose shape does not change, did not yield any difference, thus confirming the reliability of the approach.

**Conclusions:**

We have defined specific morphological features that M/M acquire in response to different acute insults by applying a sensitive and readily applicable approach to cell morphological analysis in the brain tissue. Potential application of this method can be extended to all cell types able to change shape following activation, e.g., astrocytes, or to different disease states, including chronic pathologies.

## Background

Microglia and macrophages are major cellular contributors to post-injury inflammatory response in the brain. After acute injury, rapid activation with dramatic morphological and phenotypical changes of the resident microglial cells as well as recruitment of blood-borne macrophages occurs. In the unchallenged brain, microglia constantly survey the microenvironment by extending and protruding thin branched processes [1]. After an insult, these cells readily activate and change their morphology from ramified to ameboid with enlarged soma and retracted processes [2]. These morphological changes are related to the expression of novel surface antigens and to the production of mediators that build up and maintain the inflammatory response or promote injury resolution and lesion repair [3]. At variance with microglia, infiltrated macrophages undergo only minor morphological changes during recruitment and activation [4]. Similarly to microglia, depending on the injured microenvironment, macrophages express novel/specific antigens and mediators that define their polarization state and their function [5]. It is likely that the kind of injury and its temporal and spatial evolution can drive selective microglia and macrophage activation. These cells share common myeloid origin and are often referred to as a unique population (microglia and macrophage (M/M)). It is not fully understood whether they can acquire distinct functions in the context of brain injury. Microglia and macrophages infiltrated in the injured brain are antigenically indistinguishable, and assessment of their specific function is feasible only by irradiation chimerism or parabiosis techniques [6,7].

A deeper knowledge of the M/M characteristics in relation to the nature of the brain insult and to the time and location relative to the lesion area is needed to properly manipulate the inflammatory response to promote a protective environment for therapeutical purposes [8-10]. Many aspects of M/M behavior in response to different metabolic challenges still need clarification. Notably, morphological features provide information on the functional commitment of M/M [11-13]. The ability to obtain a morphological description that may increase the insight on M/M functional meaning in brain pathology is thus a priority. M/M activation is usually measured, assessing the expression of specific M/M markers (CD11b, Iba1, F4/80) by immunohistochemistry [14]. This approach, however, does not take into consideration morphological changes that could unravel specific M/M functions after brain damage. Actin polymerization, leading to microglia shape transformations, has been demonstrated to be an ATP-dependent process [15,16]. This is at variance with what happens in macrophages that are better equipped to maintain the functionality in hypoxic/ischemic conditions [17]. Microglia are, therefore, finely tuned by the injured microenvironment that affects their shape while macrophages are characterized by their constant round-shaped morphology regardless of their tissue location.

In the present study, we provide a detailed analysis of a few morphological parameters related to M/M activation in three murine models of focal acute brain injury modeling traumatic brain injury (TBI) or stroke. Controlled cortical impact brain injury for TBI and transient or permanent occlusion of middle cerebral artery (tMCAo and pMCAo, respectively) for stroke are well established, clinically relevant models showing important functional and metabolic correlates to human pathology. A comparison of M/M morphology in the three models, each of which is characterized by different degrees of blood perfusion impairment, provides the opportunity to study M/M in different pathological scenarios, thus leading to the definition of shape descriptors that best represent the changes in M/M activation in local inflammatory events.

## Methods

### Animals

C57BL/6J wt and *cx3cr1*^+/GFP^ mice (Harlan Laboratories and Charles River, Italy) were housed in a specific pathogen-free (SPF) vivarium. Procedures involving animals and their care were conducted in conformity with institutional guidelines that are in compliance with national and international laws and policies [18]. They were reviewed and approved by the Mario Negri Institute Animal Care and Use Committee that includes ad hoc members for ethical issues and by the Italian Ministry of Health.

### Surgery protocols

#### Transient middle cerebral artery occlusion

Anesthesia was induced by 3% isoflurane inhalation in an N_2_O/O_2_ (70%/30%) mixture and maintained by 1% to 1.5% isoflurane inhalation in an N_2_O/O_2_ (70%/30%) mixture. Transient ischemia was induced by means of a siliconized filament (7-0, Doccol Corp, Redlands, CA) introduced into the right carotid artery, pushed onwards till the origin of the middle cerebral artery (MCA) and withdrawn after a 30-min ischemic period [18]. Mortality rate was 8.3%.

#### Permanent middle cerebral artery occlusion

Mice were anesthetized with 100 μl/mouse Equitensin (pentobarbital 39 mM, chloral hydrate 256 mM, MgSO_4_ 86 mM, ethanol 10% *v*/*v*, propylenglycol 39.6% *v*/*v*) administered by intraperitoneal (i.p.) injection. A vertical midline incision was made between the right orbit and tragus. The temporal muscle was excised, and the right MCA was exposed through a small burr hole in the temporal bone. The dura mater was cut with a fine needle and the MCA permanently occluded by electrocoagulation just proximal to the origin of the olfactory branch [19]. Intraoperative rectal temperature was kept at 37.0°C ± 0.5°C using a heating pad (LSI Letica). Mortality rate was 8.5%.

#### Experimental traumatic brain injury

Mice were anesthetized with 65 mg/kg sodium pentobarbital i.p. and placed in a stereotaxic frame. They were subjected to craniectomy followed by induction of TBI by controlled cortical impact [20]. This model uses a 3-mm rigid impactor driven by a pneumatic piston, rigidly mounted at an angle of 20° from the vertical plane and applied perpendicularly to the exposed dura mater over the left parietotemporal cortex at a velocity of 5 m/s and depth of 1 mm. The craniotomy was then covered with a cranioplasty and the scalp sutured. During all surgical procedures, mice were maintained at the body temperature of 37°C. No animals died after surgery.

Control mice did not receive any surgical intervention.

### Histological analysis

Twenty-four hours after surgery, mice from each group were deeply anesthetized with Equitensin (120 μl/mouse i.p.) and transcardially perfused with 30 ml of phosphate buffer saline (PBS), 0.1 mol/l, pH 7.4, followed by 60 ml of chilled paraformaldehyde (4%) in PBS. After carefully removing the brains from the skull, they were transferred to 30% sucrose in PBS at 4°C overnight for cryoprotection. The brains were then rapidly frozen by immersion in isopentane at −45°C for 3 min before being sealed into vials and stored at −70°C until use.

### Lesion size determination

Twenty-micrometer coronal brain cryosections were cut serially so to have sections distanced by ≈1 mm and stained with cresyl violet [21]. Seven slices from the bregma +2 mm to −3.76 mm were evaluated. For ischemic lesion size determination, on each slice, the infarcted area was assessed blindly and delineated by the relative paleness of histological staining tracing the area on a video screen. The infarcted area and the percentage of brain swelling for edema correction were determined by subtracting the area of the healthy tissue in the ipsilateral hemisphere from that of the contralateral hemisphere on each section [22,23]. Infarct volumes for each brain slice were quantified with computer-assisted image analyzer and calculated by Analytical Image System (Imaging Research Inc., Brock University, St. Catharines, Ontario, Canada).

For TBI contusion volume assessment, the ipsilateral and contralateral hemispheres were manually outlined. Subsequently, the injured area was calculated by subtracting the ipsilateral from the contralateral hemisphere. Finally, the contusion volume was calculated by integrating the injured area according to the previously reported formula [24].

### Immunohistochemistry

Immunohistochemistry was performed on 20-μm brain coronal sections using anti-mouse CD11b (1:800, kindly provided by Dr. Doni, [21]) and biotinylated anti-mouse CD45 (1:800, BD Biosciences Pharmigen, San Jose, CA). Positive cells were stained by reaction with 3,3 diaminobenzidine tetrahydrochloride (DAB, Vector laboratories, CA, USA). For negative control staining, the primary antibodies were omitted, and no staining was observed. CD45-positive cells displayed two morphologies [18]: a) leukocyte-like shape corresponding to cells with a rounded cell body without branches and high expression of CD45 (CD45^high^) and b) microglia-like shape having a small cell body and several branches and a fainter expression of CD45 (CD45^low^, [18]). Quantification was carried out only on CD45^high^ rounded positive cells.

### Slice selection and quantitative analysis

**Figure 1:**
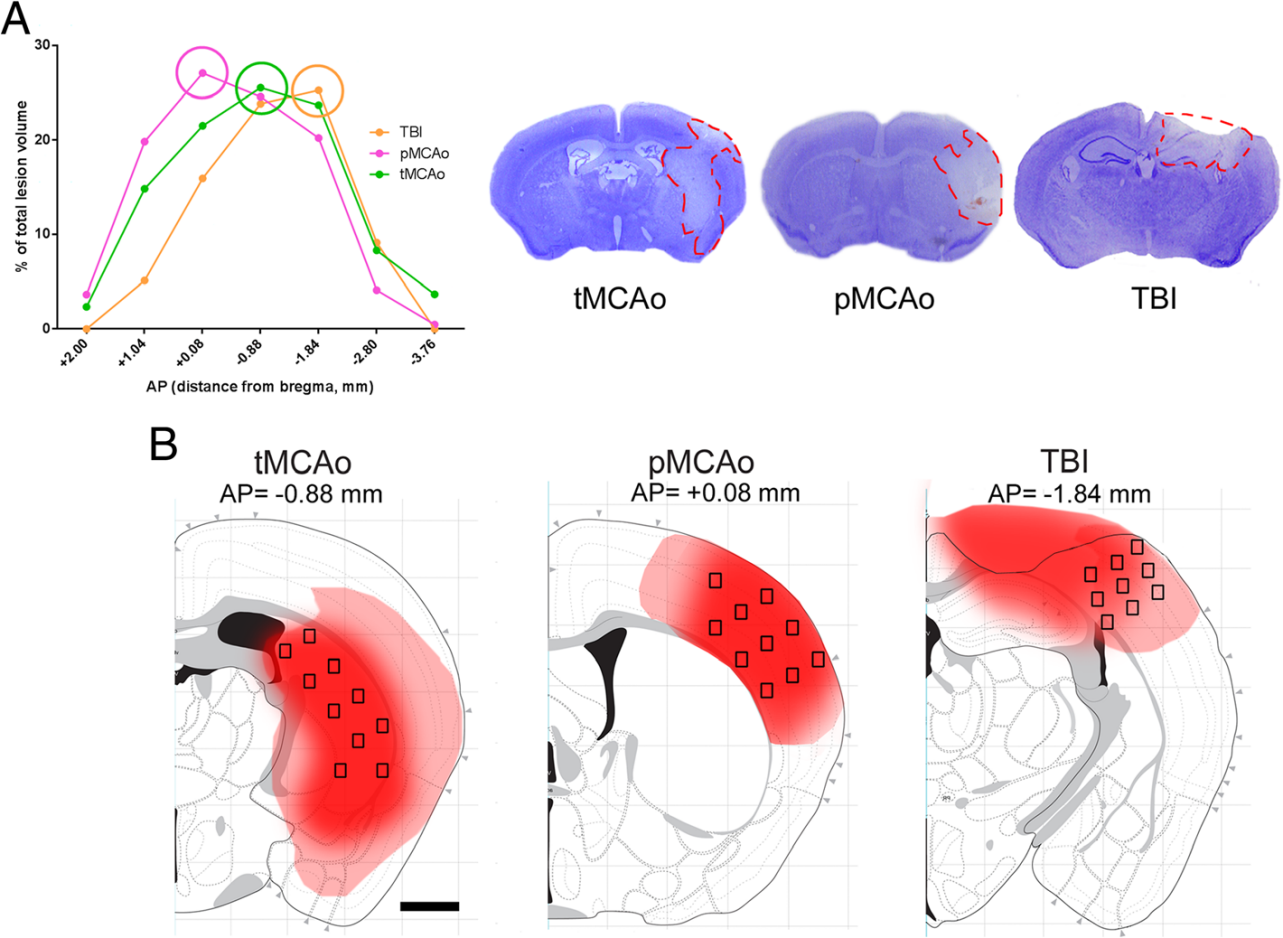
**Lesion characteristics at 24 h after tMCAo, pMCAo, or TBI and placement of acquisition fields.** Lesion size distribution is shown at different anteroposterior (AP) coordinates relative to the bregma for tMCAo, pMCAo, or TBI (*n* = 6) **(A)**. The maximum lesion size for tMCAo is at −0.88 mm from the bregma, for pMCAo at +0.08 mm, and for TBI at −1.84 mm from the bregma. For each injury model, representative sections stained by cresyl violet are shown. The lesion has been sampled by distributing ×40 acquisition fields over the region of interest as depicted in the figure **(B)** (scale bar = 1 mm). The frame centers at ×40 were distanced 532 μm (tMCAo and pMCAo) or 358 μm (TBI) for horizontally aligned frames and 266 μm for vertically aligned frames, covering 0.2 mm^2^ total area. For tMCAo and pMCAo, acquisition fields were placed within the lesion core, while for TBI, since part of the lesion core tissue is lost, acquisition fields were positioned at the contusion edge.

Marker quantification for each model was carried out over one brain coronal section per mouse at the stereotaxic coordinate showing the wider lesioned area (Figure 1A). For each model, the ischemic core was selected for quantification as follows: 1) striatum (at −0.9 mm from the bregma) that is fed by the MCA and shows the highest degree of tissue damage after its occlusion (ischemic core, [21,25]) for tMCAo; 2) cortex at 0 mm, the brain region including the ischemic core in the territory fed by the occluded branch of the MCA [19,25] for pMCAo; and 3) cortex at −1.8 mm from the bregma, where contusion edge was present (lesion core, [8]) for TBI. A drawing showing the field positioning for quantification of immunostainings within selected brain regions is depicted in Figure 1B (coordinate reference from [26]).

Field selection was performed using a BX61 Olympus microscope equipped with a motorized stage, acquiring the focal plane corresponding to image maximal sharpness (best focus) as calculated by the acquisition software AnalySIS (Olympus) [19]. Ten (for tMCAo and pMCAo) or nine (for TBI) quantification fields at ×40 magnification (pixel size = 0.172 μm) were uniformly distributed over the region of interest. Centers of frames at ×40 were distanced 532 μm (tMCAo and pMCAo) or 358 μm (TBI) for horizontally aligned frames and 266 μm for vertically aligned frames. The gap between subsequent frames was chosen to avoid overlapping acquisitions (frame centers were distanced 89.5 and 66.5 μm from frame boundaries), thus preventing cells from appearing twice in consecutive frames.

### Image processing and quantification

**Figure 2:**
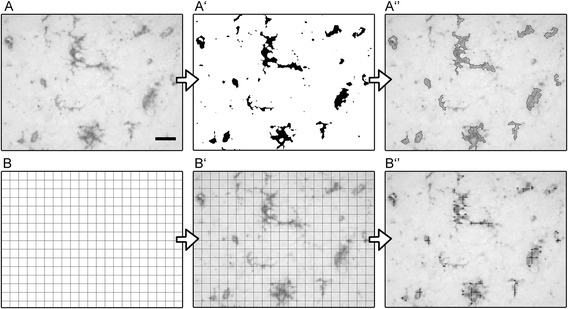
**Shape descriptor and grid crossing protocols.** Shape descriptors (area, perimeter, circularity, Feret’s diameter, aspect ratio, and solidity) were quantified on 179 × 133 μm fields **(A)**. The original image was scaled to microns and segmented by applying the Max function on Fiji (**A’**, see methods). Objects with > 25 μm^2^ area were selected and analyzed (**A’’**, outlines of selected objects for analysis). For the grid crossing counts, a grid with 9 × 9 μm spaced lines was used **(B)**. This was overimposed to the image **(B’)** and intersections between > 25 μm2 objects (previously segmented), and the grid were quantified **(B’’)**. Scale bar = 20 μm.

Image processing was performed using Fiji software [27]. An algorithm was created to segmentate and analyze stained cells. Briefly, images were first scaled into microns. Background was subtracted, and a math operation was applied so that all the gray values greater than a specified constant were replaced by the constant. The constant was defined by the operator on the basis of the best segmentation performance on pilot images and did not change across the experimental groups. Images were then binarized and smoothed to best fit cell shape and get rid of single positive pixels still present in the background. A further step of pixel erosion helped achieve satisfactory cell shape fitting. To be sure to select only cells entirely present in the acquired field, for CD11b staining, cells with an area >25 μm^2^ were analyzed (Figure 2A–A‘’). For CD45 staining, we only segmented round-shaped CD45^high^ cells, which showed more intense signal than CD45^low^ cells, by selecting appropriate max constant values. We applied a threshold of area >10 μm^2^, since the CD45 staining procedure does not use secondary antibody and results in lower signal amplification.

Once segmented, the objects meeting the minimum size to be analyzed were measured for the following parameters: area, perimeter, Feret’s diameter (caliper), circularity, aspect ratio, and solidity. Mean single cell values for each parameter were used for statistics. Segmented objects were also superimposed on a grid image with horizontal and vertical lines distanced by 9 μm. The total number of object crossing on the grid was quantified and normalized for the total number of segmented objects (Figure 2B–B‘’).

### Immunofluorescence and confocal analysis

Immunofluorescence was performed on 20-μm coronal sections according to the previously described method [18]. Primary antibodies used were anti-mouse CD45 (1:800) and anti-mouse CD11b (1:500, kindly provided by Dr. Doni). The fluorconjugated secondary antibody used was Alexa 546 anti-rat (1:500, Invitrogen, Carlsbad, CA). Appropriate negative controls without the primary antibodies were performed. None of the immunofluorescence reactions revealed unspecific fluorescent signal in the negative controls. Immunofluorescence was acquired using a scanning sequential mode to avoid bleed-through effects by an IX81 microscope equipped with a confocal scan unit FV500 with three laser lines: Ar-Kr (488 nm), He-Ne red (646 nm), and He-Ne green (532 nm, Olympus, Tokyo, Japan) and a UV diode. Three-dimensional images were acquired over a 10-μm *z*-axis with a 0.23-μm step size and processed using Imaris software (Bitplane, Zurich, Switzerland) and Photoshop CS2 (Adobe Systems Europe Ltd).

### Statistical analysis

Comparisons among groups were done by appropriate analysis of variance and post hoc test as indicated in each figure legend. Choice of parametric or non-parametric test was done after normality Kolmogorov-Smirnov test to assess whether groups met normal distribution. All groups had a normal distribution. Group size was defined by the following formula: *n* = 2*σ*2*f*(*α*,*β*) / *Δ*2 (sd in groups = *σ*, type 1 error *α* = 0.05, type II error *β* = 0.2, percentage difference between groups *Δ* = 30). Standard deviation between groups was calculated on the basis of a previous experiment for CD11b stained area quantification, where *σ* = 19, thus yielding *n* = 6.34. Statistical analysis was performed using standard software packages GraphPad Prism (GraphPad Software Inc., San Diego, CA, USA, version 6.0). All data are presented as mean and standard deviation (sd). *p* values lower than 0.05 were considered statistically significant.

## Results

A typical distribution of the lesion over the anteroposterior coordinates for each model of injury, namely, tMCAo, pMCAo, and TBI at 24 h is depicted in Figure 1A. The maximal extension of the lesioned area was located at −0.88, +0.08, and −1.84 mm from the bregma for tMCAo, pMCAo, and TBI, respectively. The lesion core corresponded to the striatum for tMCAo and to the cortex for pMCAo and TBI [8,11,19] (Figure 1B). These sections were selected for subsequent quantitative immunohistochemical analysis.

### Activated microglia (CX3CR1+ cells) show a different distribution in the three injury models 24 h after injury

**Figure 3:**
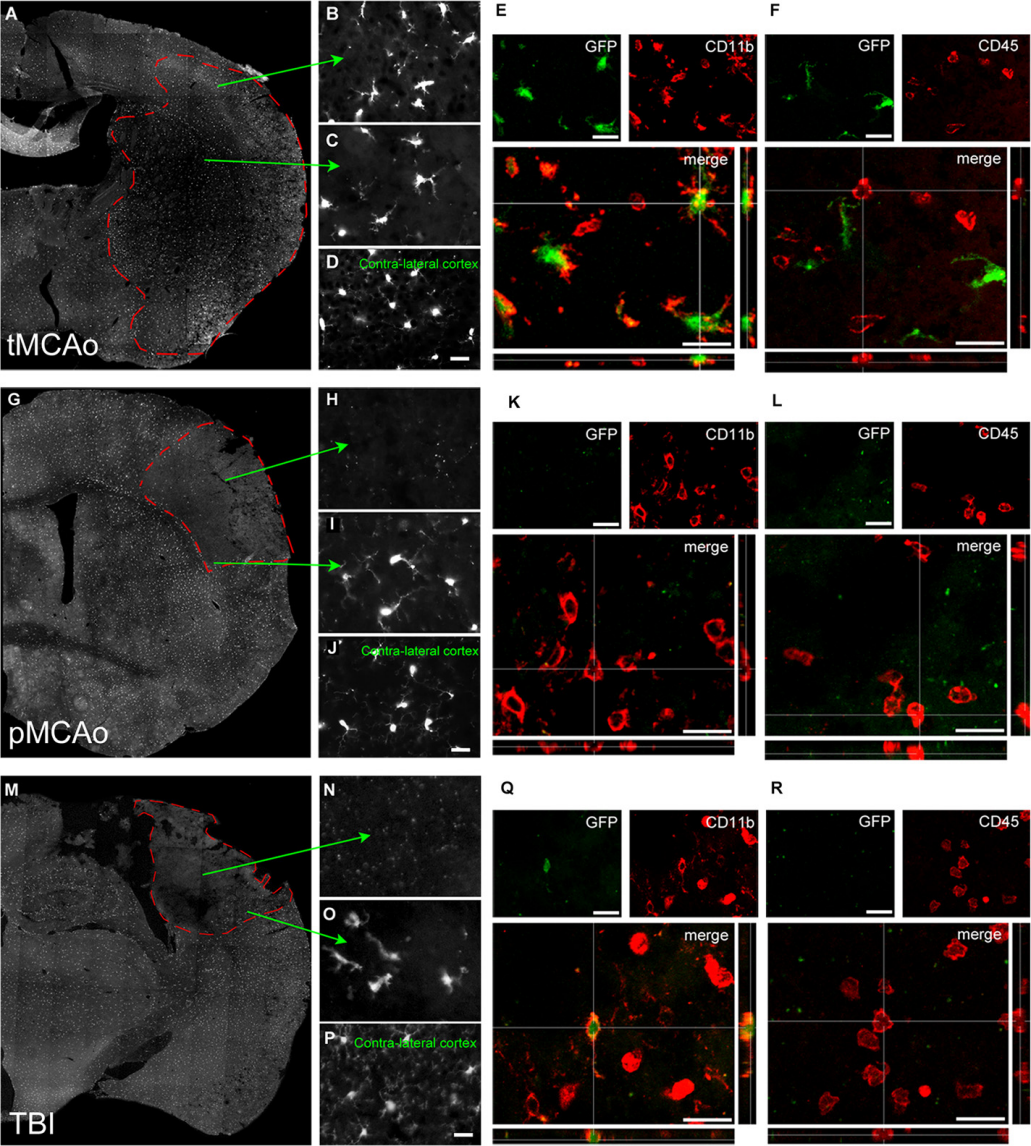
**GFP+ microglia distribution over the lesion area at 24 h after tMCAo, pMCAo, or TBI.** GFP+ microglia showed homogenous distribution over the lesioned hemisphere after tMCAo **(A)**. Microglial hypertrophic morphology was evident both in the cortex **(B)** and the striatum (lesion core, **C**). On the contralateral side GFP+ cells displayed the typical ramified morphology with thin branches **(D)**. Within the lesion core, GFP (green) co-localized with CD11b (red, pan-marker of microglia/macrophages, **E**) only in ramified CD11b+ cells. No co-localization was observed between GFP and round-shaped CD11b+ **(E)** or CD45^high^+ cells (infiltrating leukocytes, **F**). After pMCAo **(G)** GFP+ microglia were not present in the lesion core **(H)**. Hypertrophic GFP+ cells surrounded the lesion and placed at lesion border **(I)**. Normal ramified morphology was observed in contra-lateral side **(J)**. The lesion core was populated by round-shaped GFP-/CD11b+ and GFP- /CD45^high^+ cells **(K, L)**. After TBI **(M)** GFP+ microglia had similar appearance as to pMCAo: absent in the lesion core **(N)**, hypertrophic at lesion border **(O)** and ramified with thin branches in contra-lateral side **(P)**. The lesion core was populated by round-shaped GFP-/CD11b+ and GFP- /CD45^high^+ cells **(Q, R)**. Data are representative of 3 independent experiments. Scale bars= 20 μm.

*cx3cr1*^+/GFP^ mice express GFP under the control of the promoter for the fractalkine receptor (CX3CR1) that is constitutively present on microglia cell membrane, while it is inducible on macrophage subsets. GFP+ cells were found to be homogeneously distributed all over the lesion after tMCAo, displaying a hypertrophic morphology (Figure 3A–D). At variance, GFP+ cells were not detectable in the lesion core after pMCAo (Figure 3H) or TBI (Figure 3N). Following either pMCAo or TBI, hypertrophic GFP+ cells surrounded the lesion core (Figure 3I, O). Immunofluorescent stainings and confocal analysis confirmed that after tMCAo, GFP+ cells co-localized with ramified, but not round-shaped CD11b+ cells (Figure 3E), thus confirming that, in the brain parenchyma, at 24 h after injury, GFP is selectively expressed by resident microglia, as shown previously [11]. In line with this observation, GFP+ cells were negative for CD45^high^, a marker of infiltrating leukocytes (Figure 3F, [18]). In the lesion core of either pMCAo or TBI, most of the CD11b+ cells had a round-shaped morphology and did not express GFP (Figure 3K, Q). Moreover, CD45^high^+ cells present in this area did not express GFP (Figure 3L, R) similarly to what observed for the tMCAo model.

**Figure 4:**
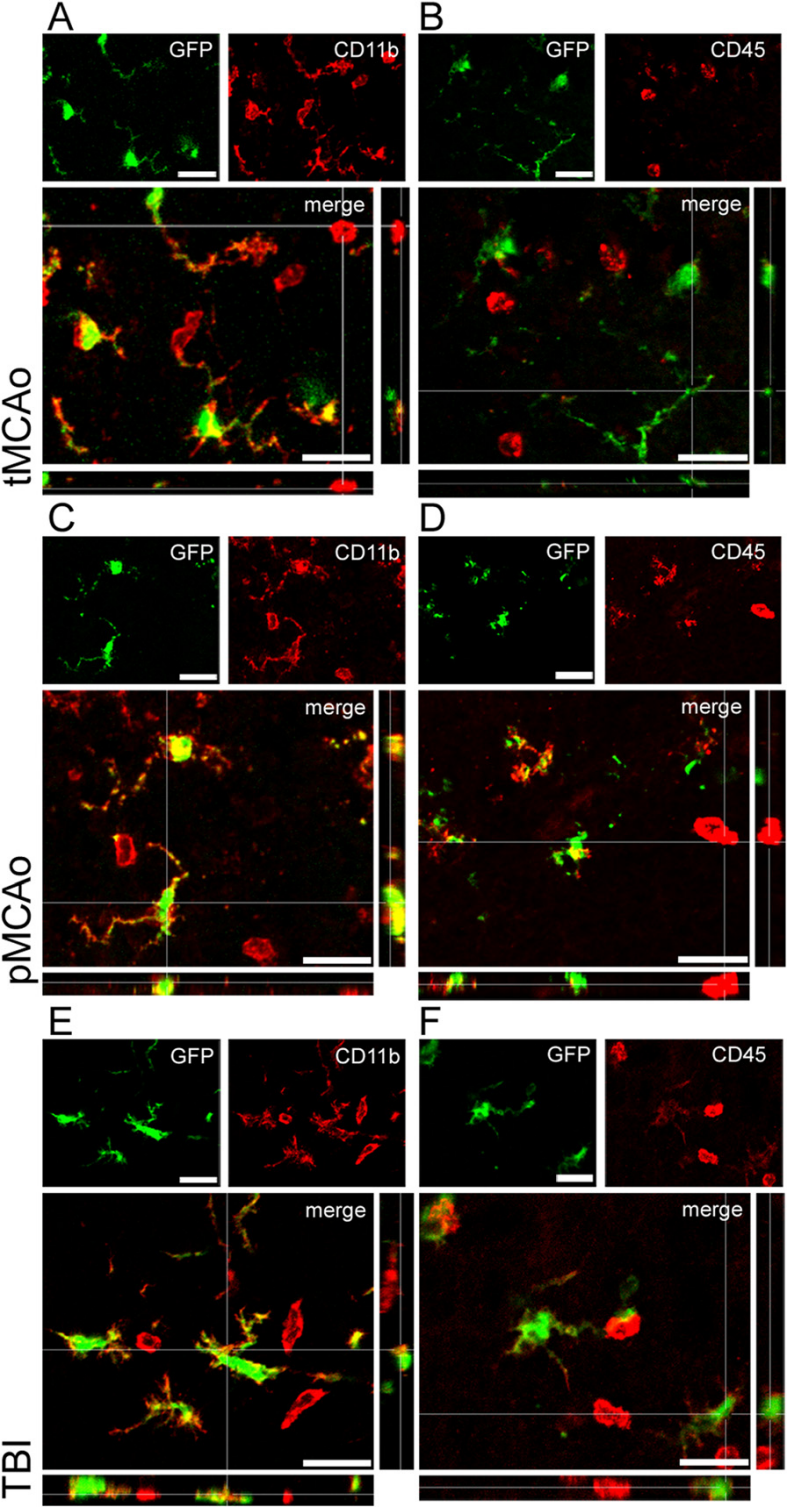
**GFP+ (green) co-localization with CD11b or CD45** ^**high**^ **(both red) in the lesion border area at 24h after tMCAo, pMCAO, or TBI.** In all tMCAo **(A)**, pMCAo **(C)** and TBI **(E)**, lesion borders were populated by ramified GFP+/CD11b+ microglia. Notably, neither round-shaped CD11b+ nor CD45^high^+ cells **(B, D, F)** expressed GFP. Data are representative of 3 independent experiments. Scale bars = 20 μm.

In the three models, GFP+ cells were present along the border zone of the lesion and co-localized with ramified, but not with round-shaped CD11b+ cells (Figure 4A, C, E) or CD45^high^+ cells (Figure 4B, D, F).

### Shape parameters for CD11b stained cells indicate a prevalence of ramified cells after tMCAo compared to either pMCAo or TBI

#### Area

**Figure 5:**
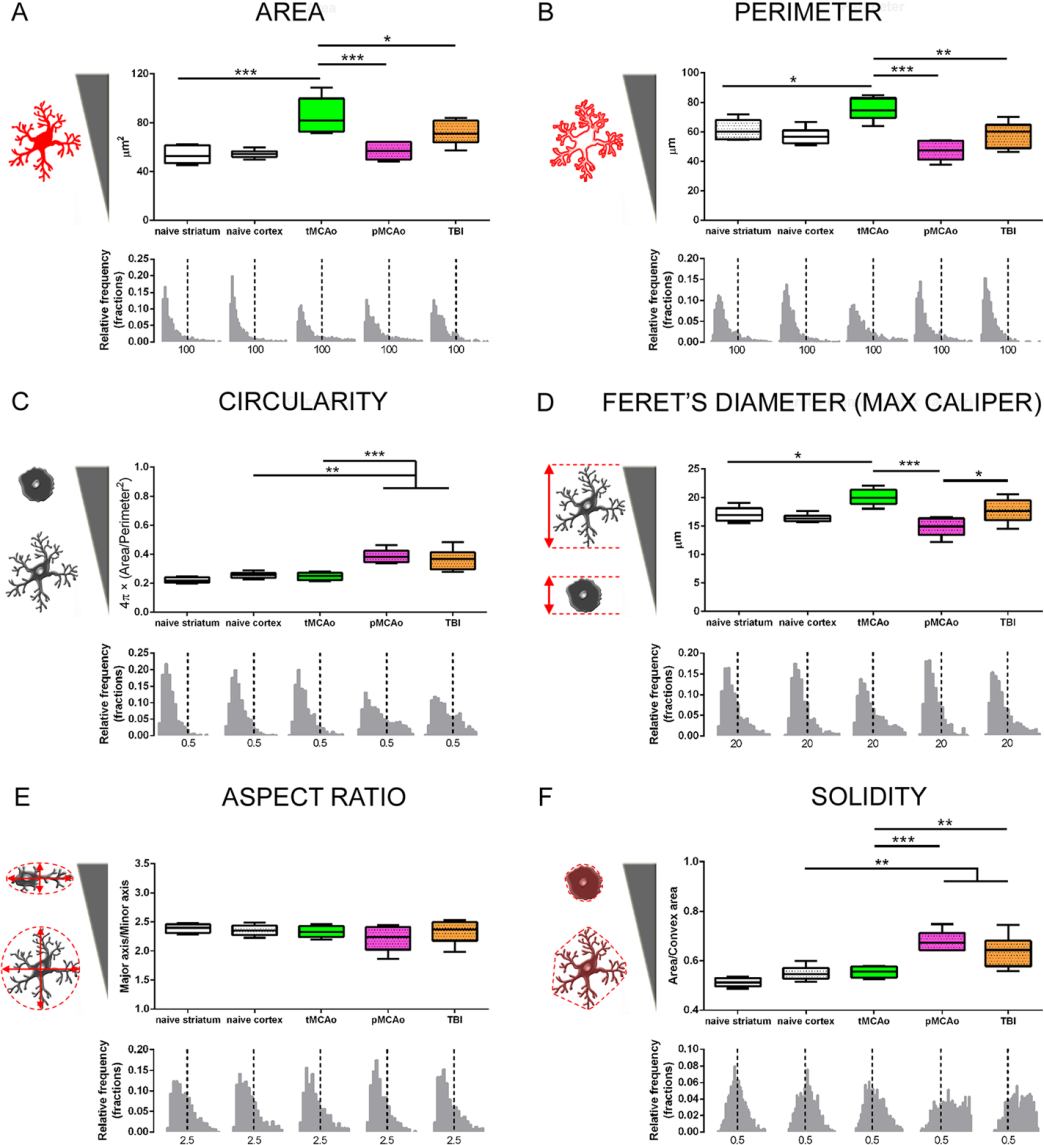
**Quantitative analysis of CD11b+ cells 24 h after acute brain injury.** Area **(A)**, perimeter **(B)**, circularity **(C)**, Feret’s diameter **(D)**, aspect ratio **(E)**, and solidity **(F)** calculated for naïve (striatum and cortex), tMCAo, pMCAo, or TBI mice are shown as box and whiskers with line at mean and min-to-max values. One-way ANOVA followed by Tukey’s multiple comparison test, *p < 0.05, **p < 0.01, and ***p < 0.001, *n* = 6. The drawings beside the *y*-axis indicate expected values for each parameter depending on cell shape (ramified vs. ameboid) **(A, B, C, D, F)** or cell symmetry **(E)**. Frequency distribution plots for individual cells are shown. Dotted lines correspond to the value indicated below each line.

Area was calculated as the total number of pixels present in a single object and is expressed in square micrometers (pixel size = 0.172 × 0.172 μm). This area is expected to increase with M/M hypertrophism due to activation, soma enlargement, and sprouting of new ramifications.

The mean area of CD11b+ cells was significantly increased after tMCAo (85.78 ± 14.60, μm^2^ ± sd) compared to naïve mice (striatum, 54.57 ± 3.33). The mean cell area after either pMCAo or TBI (57.13 ± 7.24 or 71.90 ± 9.95, respectively) was lower than that after tMCAo (Figure 5A).

#### Perimeter

This parameter was calculated based on the outline length of a given object and is expressed in microns. The perimeter is expected to be higher in activated and ramified cells.

The mean perimeter of CD11b+ cells was significantly increased after tMCAo (75.27 ± 7.56, μm ± sd) compared to naïve mice (striatum, 61.46 ± 7.38). The mean cell perimeter after either pMCAo or TBI (47.69 ± 6.90 or 58.36 ± 8.89, respectively) was lower than that after tMCAo (Figure 5B).

#### Circularity)

Circularity was calculated by the following formula: 4π × (area/perimeter^2^). This parameter varies from 0 (linear polygon) to 1 (perfect circular object).

CD11b+ cells showed higher circularity after either pMCAo (0.38 ± 0.07) or TBI (0.37 ± 0.05) compared to naïve mice (0.26 ± 0.02) and to tMCAo (0.25 ± 0.03).

By analyzing the frequency distribution plot (lower panel in Figure 5C), it appears that these differences were due to a bigger fraction of cells with >0.5 circularity in either pMCAo or TBI (Figure 5C).

#### Feret’s diameter (caliper)

Feret’s diameter represents the longest distance between two parallel lines perpendicular to that distance and drawn at the object’s boundaries. Thus, it represents the maximum caliper diameter obtained assuming that the cell was measured by means of a caliper. This parameter is expected to increase with cell hypertrophism and new ramification elongation.

CD11b+ cells in tMCAo showed increased caliper (20.10 ± 1.45, μm ± sd) compared to either naïve striatal (17.10 ± 1.35) or pMCAo cells (14.92 ± 1.70). Moreover, pMCAo had smaller caliper cells compared to TBI (17.73 ± 2.09).

In the frequency distribution plot (lower panel in Figure 5D), the fraction of cells with >20-μm caliper increased in tMCAo mice (Figure 5D).

#### Aspect ratio

Aspect ratio is calculated as the ratio between the major and the minor axis of the best fitted ellipse to a given object. This parameter varies with cell morphological polarization, being higher in elongated cells (Figure 5E).

No statistically significant differences could be detected in this parameter.

#### Solidity

Solidity is calculated by dividing the area of an object by its convex area. The latter is the area of the smallest polygon drawn around the object that satisfies two conditions: 1) it is convex (all its interior angles measure less than 180°) and 2) it contains the original region. The more ramified is the object, the bigger is the convex area, thus yielding a smaller solidity (Figure 5F).

CD11b+ cells in either pMCAo or TBI mice displayed higher solidity (0.68 ± 0.04 or 0.65 ± 0.03, respectively) compared to controls (naïve cortex, 0.55 ± 0.03) and to tMCAo (0.55 ± 0.02).

An increase in the fraction of cells with >0.5 solidity was evident for either pMCAo or TBI (lower panel in Figure 5F).

#### Grid crossings

**Figure 6:**
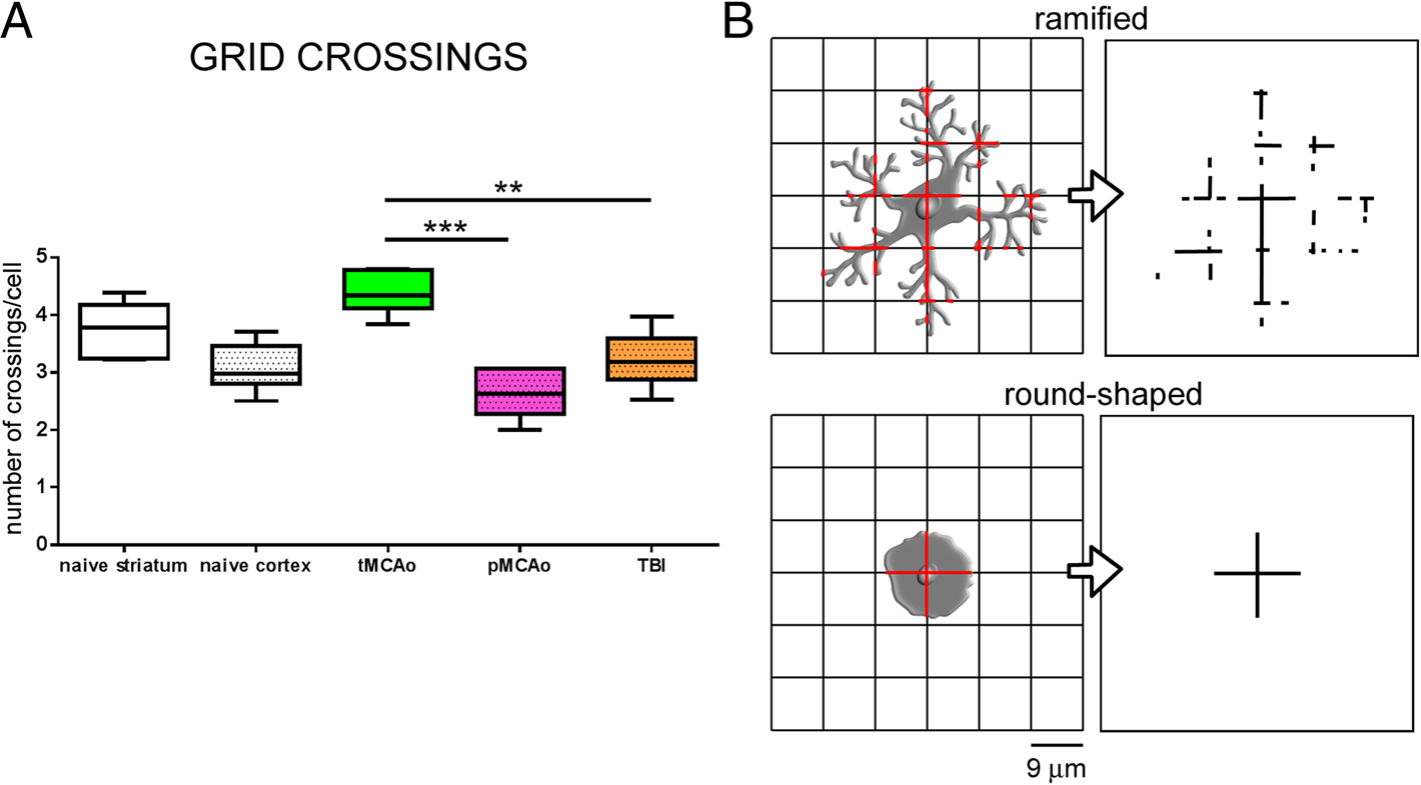
**Grid crossings by CD11b stained cells 24h after acute brain injury.** CD11b+ cells in the lesion core displayed significantly increased grid crossings after tMCAo compared to either pMCAo or TBI **(A)**. This indicates an increased presence of ramified microglia in tMCAo, since ramified cells are expected to have more frequent crossings with the grid compared to round-shaped cells **(B)**. Data are shown as box and whiskers with line at mean and min-to-max values. One-way ANOVA followed by Tukey’s multiple comparison test, **p<0.01, ***p<0.001, n=6.

When a grid with 9 μm spaced lines is overimposed to an image of stained cells, the number of crossing between the stained objects (>25 μm^2^ area) and the grid can be calculated (Figure 6A). Total number of crossings is then normalized to the total number of objects. Ramified objects are expected to have frequent crossings with the grid (Figure 6B, top panel), while round-shaped ones have few crossings (Figure 6B, bottom panel). Cells in tMCAo crossed the grid 4.39 ± 0.36 times (mean ± sd), significantly more frequently than pMCAo (2.66 ± 0.44) or TBI (3.22 ± 0.49) cells.

### Shape parameters for CD45^high^ cells had constant values in the three models

**Figure 7:**
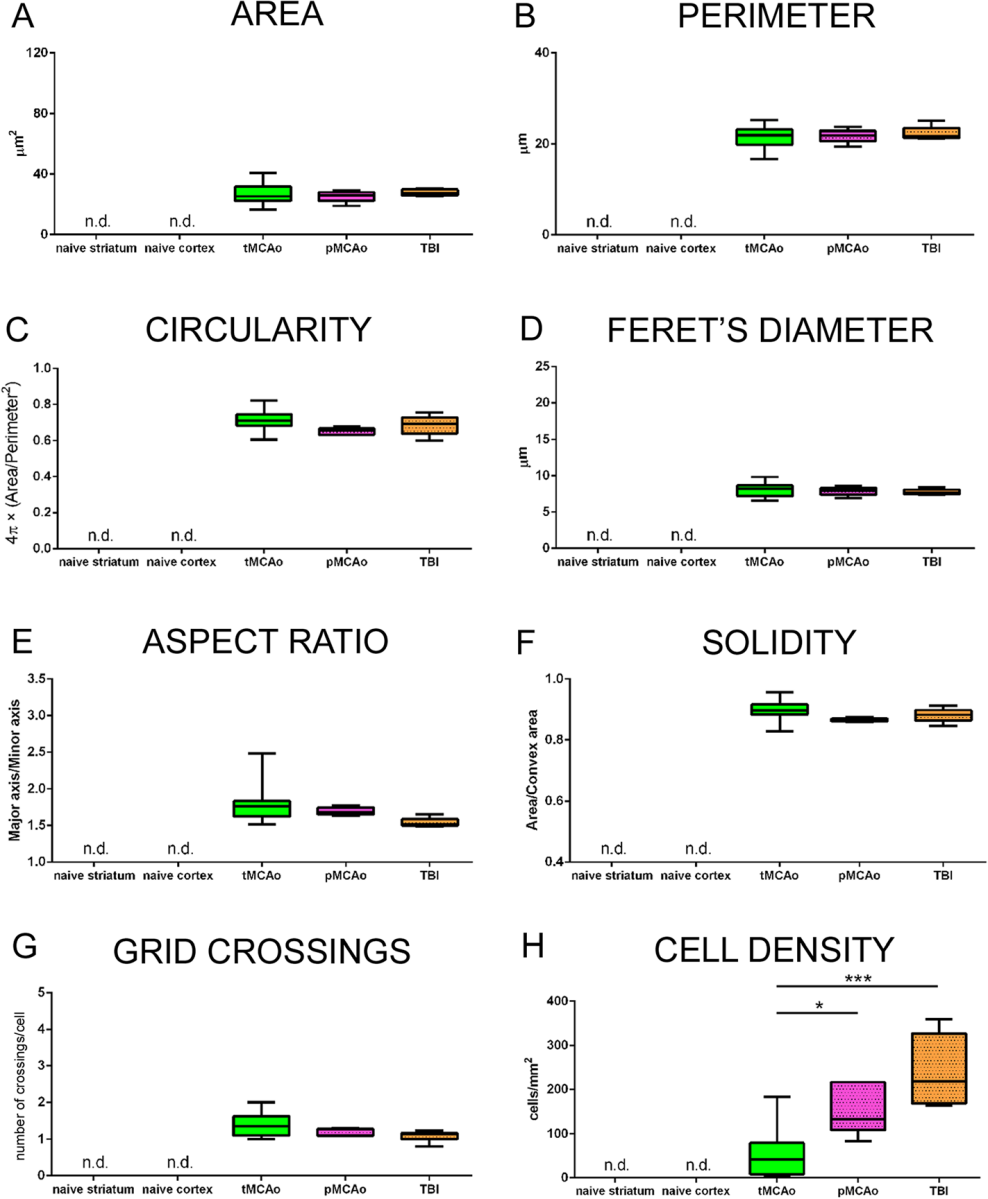
**Quantitative analysis of CD45** ^**high**^ **+ cells 24 h after acute brain injury.** Area **(A)**, perimeter **(B)**, circularity **(C)**, Feret’s diameter **(D)**, aspect ratio **(E)**, solidity **(F)**, and grid crossings **(G)** calculated for naïve (striatum and cortex), tMCAo, pMCAo, or TBI mice are shown as box and whiskers with line at mean and min-to-max values. None of these parameters changed across models, in line with the unvaried round-shaped morphology of CD45^high^+ cells. CD45^high^+ cell density **(H)** in the lesion core after pMCAo and TBI was higher compared to tMCAo. One-way ANOVA followed by Tukey’s multiple comparison test, *p < 0.05, ***p < 0.001, *n* = 6 (n.d. = not detectable).

The previously reported shape parameters were applied to CD45^high^+ cells. These are the round-shaped cells that are recruited to the brain after acute injury [8,11,19] and that are not prone to change morphology. As expected, none of the shape parameters tested showed a significant difference in the three injury models (Figure 7A–F). In naïve mice, we could not detect any CD45^high^+ cells, consistently with no immune cell infiltration in unpeturbed brain tissue [18,19].

Grid crossings did not vary among the groups (Figure 7G) and the values were below 1.5 crossing per cell, thus indicating the presence of small round objects (see Figure 6B).

In line with an increased presence of round-shaped cells in pMCAo or TBI (see Figure 5A, F), these lesions showed an increased infiltration of CD45^high^+ cells (156.70 ± 58.45 or 240.90 ± 81.82, cells/mm^2^ ± sd, respectively) compared to tMCAo (50.76 ± 54.38, Figure 7H).

## Discussion

In the present study, we have determined the morphology of brain CD11b cells, defining specific population subsets present in the injured tissue following different injurious stimuli. Specifically, we have validated objective parameters associated with morphology, including area, perimeter, Feret’s diameter (caliper), circularity, aspect ratio, solidity, and the number of grid crossings between cell-positive area and a grid.

We used three models of acute brain injury, namely, tMCAo, pMCAo, and TBI, that share blood perfusion deficits and metabolic derangements as common events, and we described distinctive morphological features of recruited/activated M/M in the injured tissue. We showed that the shape descriptors that best represent M/M ramification/elongation are area and perimeter, while circularity and solidity provide information on the ameboid shape. We also provided preliminary evidence on the involvement of different populations in local inflammatory events, with macrophages replacing microglia cells into the lesion core in case of no reperfusion.

### CX3CR1 (fractalkine receptor) is expressed exclusively by resident microglia in brain parenchyma 24 h following acute injury

Appropriate discrimination between microglia and macrophages would help define whether or not these cells, although sharing multiple features [3,28], have selective roles during neuroinflammation. We here show that the expression of GFP in *cx3cr1*^GFP/+^ mice allows to discriminate between microglia and macrophages at early time points after acute insult (24 h). In a previous work, we demonstrated that, after tMCAo, GFP is expressed by ramified resident microglia, while infiltrating immune cells (round-shaped CD11b+, CD45^high^+, and CD3+) do not express it [11]. We now extend the same observation to pMCAo and TBI, both conditions inducing immune cell infiltration and microglia activation at this early stage [8,19,20,29]. The first subset of infiltrating macrophages is early recruited via the CCR family receptors and do not express CX3CR1 [30-32].

### Resident microglia are depleted if no reperfusion is allowed

It is generally believed that, once infiltrated in the brain, macrophages populate the same lesioned areas as microglia. However, the distinct resilience of macrophages and microglia to ATP loss may cause different distribution of these populations over the lesioned area. In the case of ischemic/reperfusion injury, both microglia and macrophages are present in the lesion core. In the case of no reperfusion, e.g., pMCAo—lesion core, TBI—contusion edge, a large area depleted from microglia is consistently detected. In our study, the microglia-empty territory was replenished by round-shaped CX3CR1−/CD11b+/CD45^high^+ cells, which are likely to be the infiltrating macrophage population. After selective microglia depletion in *CD11b-HSVTK* mice [33], macrophages engraft microglia-depleted regions and acquire a microglia-like behavior, extending processes towards an ATP source and keeping the cerebral homeostasis.

In case of a severe acute injury with impairment of cerebral blood flow (CBF), brain homeostasis is disrupted and metabolic crisis occurs, leading to massive death of cerebral populations, including microglia. In these conditions, macrophages, which can switch to anaerobic metabolism [17], infiltrate and acquire specific functional phenotypes that evolve with the lesion progression [19]. A different behavior of macrophages compared to microglia may be observed, being microglia mostly located at pMCAo or TBI lesion boundaries, with lower expression of polarization markers [19]. Additional studies are needed to relate features of microglia and macrophages in acute brain injury to specific function.

### CD11b is a reliable marker to study M/M morphology

We selected M/M on the basis of CD11b positivity [19,29]. Due to its presence on the membrane surface and to its constitutive expression on myeloid cells, CD11b is particularly suitable to study the morphology of M/M in either physiological or pathological conditions. M/M are often labeled by using other constitutive markers, e.g., Iba1 and CD45, or by isolectin B4 from *Griffonia semplicifolia* that binds α-d-galactosyl residues. Among these markers, only Iba1 provides information comparable to those obtained with CD11b on M/M activation and morphology [14]. Indeed, CD45 yields a weak signal in low expressing cells, e.g., microglia, whose identification might be tricky, so this marker is best used for CD45^high^+ cell counting [11,18,19], while isolectin B4 shows non-homogeneous labeling of M/M and binds also to endothelial cells [34].

### M/M morphological features 24 h after injury indicate differential M/M activation and subtype distribution depending on the kind of injury

CD11b+ cell area and perimeter were higher in tMCAo compared to naïve animals (striatum), indicating the presence of hypertrophic microglia, as well as to pMCAo and TBI mice, due to the increased infiltration of CD45^high^+ cells in these latter models. Infiltrated macrophages are indeed smaller than hypertrophic microglia and with limited ability to change morphology, thus explaining the smaller CD11b+ cell size found in pMCAo or TBI.

Interestingly, CD11b+ cell Feret’s diameter in TBI was bigger than that in pMCAo. In TBI, most of the lesion core is lost and sampling included wider border areas where hypertrophic microglia sit. We did not detect significant differences in area, perimeter, and Feret’s diameter between pMCAo or TBI and naïve cortex. Steady-state microglia in naïve mice have thin ramifications that, due to the microscope resolution limit, could have been excluded from the identified objects. Moreover, the bidimensional acquisition of the images fails to properly include ramifications belonging to different focal planes. These technical limits could lead to underestimation of area, perimeter, and Feret’s diameter in steady-state microglia of naive animals, resulting in a lack of sensitivity. It should be mentioned that naïve and not sham-operated animals were used in this study potentially raising the issue on the surgery versus injury effect. In previous works, we constantly observed M/M activation based on CD11b immunoreactivity, by comparing each model with proper sham-operated mice, thus confirming an injury effect and showing negligible impact of the surgical procedure on cortical and striatal M/M activation ([11] for tMCAo; [19] for pMCAo; unpublished submitted data for TBI). The focus of the present study was the comparison of M/M morphology in the three models and we choose an unperturbed condition as a common control group.

A clear-cut difference between pMCAo/TBI and naive animals was obtained measuring circularity and solidity. These parameters yielded similar results and may be considered as indices of the same morphological feature. CD11b+ cells showed a more round-shaped morphology in pMCAo and TBI, in line with increased infiltration of peripheral immune cells.

Aspect ratio was the only parameter not changing across the models. It indicates the morphological polarization of a given object. The observation that in tMCAo aspect ratio did not change compared to pMCAo, or TBI, suggests that at 24 h CD11b+ cells were not directed towards a specific site. Directional ramification extension may be more relevant at earlier time points due to the damage-sensing microglia reorientation. At 24 h, microglia already show a hypertrophic ameboid morphology characterized by short and numerous processes, symmetrically extending from the cell soma [11].

Microglia morphology can also be influenced by the cellular architecture of specific brain areas [35]. The observed symmetric morphology may be due to the sampling of the gray matter, where microglia extend processes radially [36]. At variance with this, the white matter is populated by a specific type of microglia with bipolar, elongated morphology, called rod microglia. Rod microglia are in close association with neurons [37,38] and take part to axonal damage and recovery after injury [39].

### The grid crossing method offers a reliable measure of M/M morphology

This technique is usually applied to quantify vascular density [40]. In the context of CD11b+ morphology evaluation, the number of grid crossings can be used to provide information on ramification density, being ramified cells able to cross the grid more frequently than round-shaped objects. In line with this, tMCAo mice had increased grid crossings compared to either pMCAo or TBI. This approach may be considered a simplified version of the Sholl analysis which, based on single cell selection and overimposition to a field formed by concentric circles with increasing radius, provides quantification of branch length and frequency [41]. Although accurate and applicable to measure microglia morphology [11], Sholl analysis requires single cell selection, thus limiting the total number of quantified cells (small region of interest). The method of grid crossings proposed here can instead be applied to big image data sets, allowing to obtain measures over wide tissue portions in a limited time. The sensitivity may be appropriately enhanced by changing the grid dimensions.

### Validation of the proposed approach

The observation that CD45^high^+ cells, whose shape is not expected to change, did not show significant changes in the morphological parameters further confirmed the validity of the proposed approach. None of the shape descriptors used, neither the grid crossing count, was different in the three models, as expected. CD45^high^+ cell density was greater in pMCAo or TBI compared to tMCAo, in line with increased immune cell infiltration in injury models with no reperfusion [42,43] compared with ischemic/reperfusion injury [44].

## Conclusions

In conclusion, we obtained a full characterization of morphological M/M state, by validating quantitative parameters, over wide injured tissue portions. We showed that only when reperfusion is there, microglial cells are present in the injured core where they become activated. At variance with this, when reperfusion is not allowed, microglial cells populate the area surrounding the lesion core, and this latter is in turn replenished by infiltrating macrophages. These pieces of information set the line to clarify the functional meaning of M/M activation and potential distinctive features of microglia versus macrophages in injury resolution and repair.

We provide a sensitive and readily applicable approach for morphological analysis of stained objects with the intrinsic limitation related to the bidimensional approaches. We used appropriate size thresholds to exclude small objects not representing entirely a cell, such as ramification portions from out-of-focus cells, and we distributed acquisition frames equally by an operator non-dependent sampling of the region of interest, thus limiting the impact of non-stereological acquisition.

Notably, the (potential) strength of our approach goes beyond the proposed application since in principle it can be applied to all cell types able to change shape following activation, e.g., astrocytes, or to different disease states, including chronic pathologies.

## Abbreviations

CBF: Cerebral blood flow
DAB: 3,3 Diaminobenzidine tetrahydrochloride
GFP: Green fluorescent protein
i.p.: Intraperitoneal
MCA: Middle cerebral artery
M/M: Microglia and macrophage
n.d.: Not detectable
PBS: Phosphate buffer saline
pMCAo: Permanent middle cerebral artery occlusion
sd: Standard deviation
TBI: Traumatic brain injury
tMCAo: Transient middle cerebral artery occlusion
